# A matter of concern – Trace element dyshomeostasis and genomic stability in neurons

**DOI:** 10.1016/j.redox.2021.101877

**Published:** 2021-01-24

**Authors:** Viktoria K. Wandt, Nicola Winkelbeiner, Julia Bornhorst, Barbara Witt, Stefanie Raschke, Luise Simon, Franziska Ebert, Anna P. Kipp, Tanja Schwerdtle

**Affiliations:** a Department of Food Chemistry, Institute of Nutritional Science, University of Potsdam, Arthur-Scheunert-Allee 114-116, 14558, Nuthetal, Germany; b TraceAge – DFG Research Unit on Interactions of Essential Trace Elements in Healthy and Diseased Elderly (FOR 2558), Berlin-Potsdam-Jena-Wuppertal, Germany; c Food Chemistry, Faculty of Mathematics and Natural Sciences, University of Wuppertal, Gaußstr. 20, 42119, Wuppertal, Germany; d Department of Molecular Nutritional Physiology, Institute of Nutritional Sciences, Friedrich Schiller University Jena, Dornburger Str. 24, 07743, Jena, Germany; e German Federal Institute for Risk Assessment (BfR), Max-Dohrn-Str. 8-10, 10589, Berlin, Germany

**Keywords:** Trace elements, Trace element homeostasis, Brain, DNA damage (response), Base excision repair, Genome stability

## Abstract

Neurons are post-mitotic cells in the brain and their integrity is of central importance to avoid neurodegeneration. Yet, the inability of self-replenishment of post-mitotic cells results in the need to withstand challenges from numerous stressors during life. Neurons are exposed to oxidative stress due to high oxygen consumption during metabolic activity in the brain. Accordingly, DNA damage can occur and accumulate, resulting in genome instability. In this context, imbalances in brain trace element homeostasis are a matter of concern, especially regarding iron, copper, manganese, zinc, and selenium. Although trace elements are essential for brain physiology, excess and deficient conditions are considered to impair neuronal maintenance. Besides increasing oxidative stress, DNA damage response and repair of oxidative DNA damage are affected by trace elements. Hence, a balanced trace element homeostasis is of particular importance to safeguard neuronal genome integrity and prevent neuronal loss. This review summarises the current state of knowledge on the impact of deficient, as well as excessive iron, copper, manganese, zinc, and selenium levels on neuronal genome stability.

## Highlights

• Post-mitotic neurons show an increased vulnerability to oxidative stress.
• Trace element dyshomeostasis impairs neuronal genome maintenance, affecting DNA damage response as well as DNA repair.
• The review summarises the effects of excessive and deficient trace element levels neuronal genome stability maintenance.

## Graphical abstract

**Figure:**
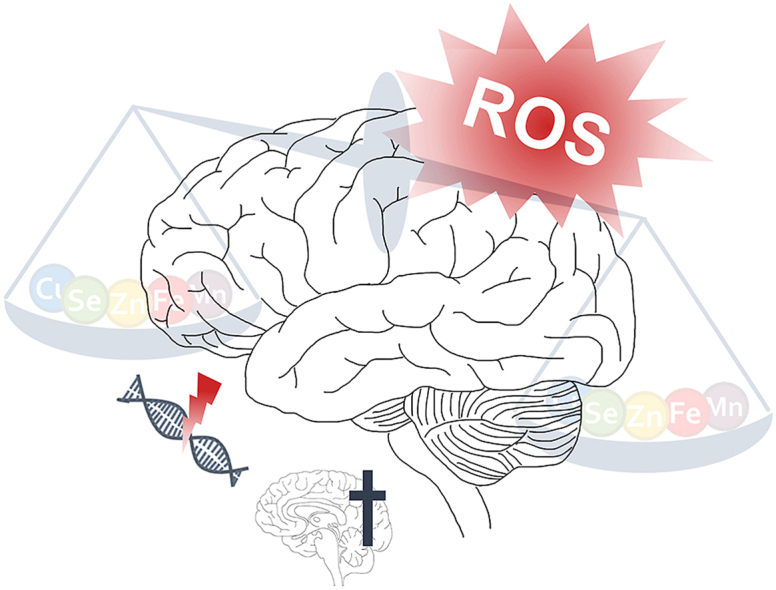

## 1 Introduction

The brain consumes 20% of the total basal oxygen budget in human body to maintain physiological brain function, among others for adenosine triphosphate (ATP) production via oxidative phosphorylation in mitochondria [[1], [2], [3]]. Within the respiratory chain, free radicals as well as non-radical reactive species, notably superoxide anion, hydroxyl radical, and hydrogen peroxide, accumulate as byproducts. While a controlled production of these reactive oxygen species (ROS) is necessary for optimal brain function, such as for redox signalling, an accumulation, exceeding the antioxidant scavenging ability of the cell, is linked to pathophysiological changes associated with neurodegenerative diseases and ageing [1,3]. In the brain, a multitude of interrelated factors exist that increase the vulnerability of neuronal cells to experience oxidative damage [3]. Neurons are enriched in mitochondria and possess a rather high metabolic turn-over, resulting in ROS production in the respiratory chain [4]. Furthermore, high concentrations of unsaturated fatty acids in neuronal cell membranes increase the probability of their oxidation resulting in lipid peroxidation [5]. This is possible because neuronal cells only have a modest endogenous antioxidant system. Both antioxidant enzymes and low molecular weight antioxidants exhibit a low concentration in the brain compared to other organs [6,7]. Cytosolic GSH content is approximately 50% lower in neurons compared to hepatocytes. In this context, GSH-linked enzyme systems like glutathione peroxidase (GPX) 4 and peroxiredoxin 6 may show a restricted activity, increasing the risk of hydrogen peroxide accumulation, and hence elevate the vulnerability of neuronal cells to a disturbed redox homeostasis [8,9]. Further key factors include neurotransmitter autoxidation [3], as well as the abundance of redox-active transition metals, such as iron (Fe), copper (Cu), and manganese (Mn). Increasing concentrations of these transition metals can result in increased hydroxyl radical formation via Fenton or Fenton-like mechanisms [10]. Resulting ROS accumulation can have profound, negative consequences in particular in non-self-replenishing post-mitotic neuronal cells, causing oxidative damage, cellular degeneration, and even functional decline [2,11,12]. Besides extensive protein and lipid peroxidation, especially DNA is prone to ROS attack due to its limited chemical resilience, eventually resulting in genomic instability [13,14]. Consequently, DNA damage response (DDR) as well as DNA repair pathways play a pivotal role in safeguarding neuronal cell maintenance.

In this context, not only imbalances in redox-active trace elements (TEs) Fe, Cu, and Mn, but also imbalances in other essential TEs, such as zinc (Zn) and selenium (Se), can have an impact on the generation of oxidative stress in post-mitotic neuronal cells, as well as on a multitude of pathways involved in antioxidant systems, DDR, and DNA repair [10,[15], [16], [17], [18]]. While dyshomeostasis in the redox-active TEs Fe and Cu facilitates the generation of oxidative stress, dyshomeostasis in TEs involved in antioxidant systems such as Zn, Se, and Mn weakens the cellular capacity to defend ROS [[15], [16], [17], [18]]. Hence, besides their pivotal role in multifunctional biochemical and physiological processes in the brain, such as the involvement in neuronal cellular regulation and survival as well as their impact on neurogenesis [19,20], alterations in TEs are strongly associated with impaired neuronal functions [21,22]. Therefore, a maintained trace element (TE) homeostasis is crucial [19,20]. Furthermore, alterations in special TE homeostases are strongly associated with impaired neuronal function, contributing among others to the development of neurodegenerative diseases like Alzheimer's (AD) and Parkinson's disease (PD). Also, systemic TE dyshomeostasis, as for example for Cu occurring in Wilson's disease (WD), is characterized by marked neurological impairment [21,22].

**Fig. 1:**
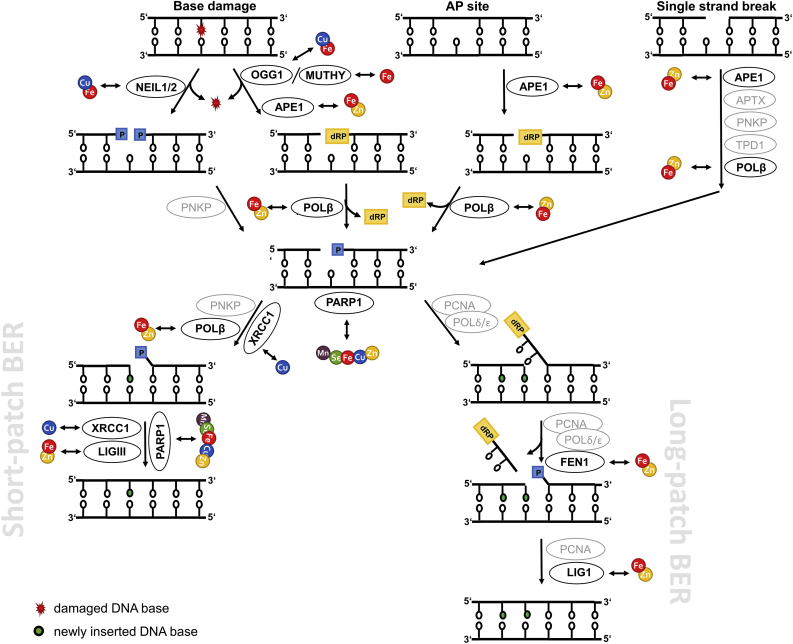
**Simplified scheme illustrating mammalian short- and long-patch BER.** The figure highlights the key BER enzymes and their interactions with the TEs Fe, Cu, Mn, Zn, and Se in neuronal cells summarized in this review. In this context, by TE dyshomeostasis affected enzymes are illustrated in black, whereas other important enzymes are highlighted in grey. APE1 = apurinic/apyrimidinic endonuclease 1; APTX = aprataxin; FEN1 = flap structure-specific endonuclease 1; LIG1/3 = ligase 1/3; MUTYH = mutY DNA glycosylase; NEIL1/2 = nei like DNA glycosylase 1/2; OGG1 = 8-oxoguanine DNA glycosylase; PARP1 = poly(ADP-ribose)polymerase 1; PARylation = poly(ADP-ribosyl)ation; PCNA = proliferating cell nuclear antigen; PNKP = polynucleotide kinase 3′-phosphatase; POLβ = DNA polymerase beta; POLδ/ε = polymerase δ/ε; TPD1 = tyrosyl-DNA phosphodiesterase 1; XRCC1 = X-ray repair cross complementing 1.

To safeguard the cellular genome from (oxidative) damage, especially in post-mitotic neurons which incur DNA damage over a long time, DDR and DNA repair mechanisms are essential. In response to DNA damage, one of the earliest cellular events is activation of the complex DDR signal transduction pathway [23]. With the aim to maintain genomic integrity, this tightly regulated machinery of sensor, transducer, and effector proteins coordinates DNA repair, induces cell cycle arrest to provide time for repair, and can also initiate apoptosis or cellular senescence to limit the DNA damage extent. Prominently involved in exerting these tasks is the tumour suppressor protein p53, the so-called “guardian of the genome” [24]. However, in post-mitotic cells, cell cycle relevant genes are permanently silenced. This includes downregulation of genes related to DDR, such as *TP53* encoding for p53 [12]. Following DDR activation in response to oxidative DNA damage, base excision repair (BER) as the major repair pathway for small, often ROS-derived DNA lesions, apurinic/apyrimidinic (AP) sites, and single-strand breaks is initiated [1,25,26]. Depending on the origin and chemical structure of single-strand breaks, the single-strand break repair pathway can be initiated, which is closely related to BER and involves additional and BER repair factors [27]. Furthermore, excessive ROS can result in the formation of double-strand breaks via induction of lesions and single-strand breaks in close proximity, which are repaired via non-homologous endjoining in post-mitotic neurons [28,29]. BER proceeds through five steps: (i) damage recognition and removal by a specific mono- or bifunctional DNA glycosylase; (ii) incision at the resulting AP site either by the bifunctional glycosylase itself or, for monofunctional glycosylases, by an AP endonuclease; (iii) endprocessing of the produced blocked termini of the gap; (iv) gap-filling by DNA polymerases; and (v) ligation of the damaged DNA strand by a DNA ligase. For step (ii), depending on the number of excised nucleotides, BER is subdivided into the predominantly executed short-patch and the long-patch BER pathways, which require a different set of repair enzymes for differing damaged substrates. A simplified scheme of BER including TE interactions with involved repair enzymes is illustrated in Fig. 1 and an overview of DDR and BER proteins targeted by the TEs considered in this review is provided in Table 1. BER, maintenance of genomic stability, and DDR enzymes form an intricate network with enzymes not only fulfilling single functions in DDR or BER, but showing overlapping modes of action. For example, poly(ADP-ribose)polymerase (PARP) 1 contains three zinc-finger motives, thus is susceptible to changes in Zn homeostasis, and acts as damage sensor within the DDR but is also involved in BER [25,30].

**Table 1:** **Important mammalian key players in BER, maintenance of genome stability, and DDR targeted by alterations in brain Fe, Cu, Mn, Zn, and Se levels.** APE1 = apurinic/apyrimidinic endonuclease 1; ATM = ataxia telangiectasia mutated serine/threonine kinase; FEN1 = flap structure-specific endonuclease 1; LIG1/3 = ligase 1/3; MUTYH = mutY DNA glycosylase; NEIL1/2 = nei like DNA glycosylase 1/2; OGG1 = 8-oxoguanine DNA glycosylase; p53 = tumour protein p53; PARP1 = poly(ADP-ribose)polymerase1; PARylation = poly(ADP-ribosyl)ation; POLβ = DNA polymerase beta; XRCC1 = X-ray repair cross complementing 1.

Enzyme	Impacting TE	Effect concentration	
Base excision repair			
APE1	Fe ↑ (activity ↓)	≥10 μM FeCl_3_ (rodent brain nuclear extract, 5 min)	[51]
	Zn ↓ (expression ↑)	(C6 rat glioma cells, grown in media rendered Zn-deficient)	[17]
FEN1	Fe ↑ (activity ↓)	≥10 μM FeCl_3_/≥ 50 μM FeSO_4_ (human recombinant FEN1, 5 min); ≥250 μM FeCl_3_ (rodent brain nuclear extract, 5 min)	[51]
	Zn ↑ (activity ↓)	≥10 μM ZnCl_2_ (human recombinant FEN1, 5 min); ≥250 μM ZnCl_2_ (rodent brain nuclear extract, 5 min)	
LIG1/3	Fe ↑ (activity ↓)	≥10 μM FeCl_3_ (rodent brain nuclear extract, 5 min)	[51]
	Zn ↑ (activity ↓)	≥10 μM ZnCl_2_ (rodent brain nuclear extract, 5 min)	
MUTYH	Fe ↑ (expression ↓)	5000 mg/L ferric ammonium citrate (C57BL/6J, 3 month)	[44]
NEIL1/2	Cu ↑ (activity ↓)	50 μM CuCl_2_ (SH-SY5Y cells, 24 h, twice)	[52]
	Fe ↑ (activity ↓)	100 μM FeSO_4_ (SH-SY5Y cells, 24 h, twice)	
OGG1	Cu ↑ (expression ↑)	350 μM CuSO_4_ (SH-SY5Y cells, 24 h)	[92]
	Fe ↑ (expression ↓)	5000 mg/L ferric ammonium citrate (C57BL/6J, 3 month)	[44]
POLβ	Fe ↑ (activity ↓)	≥10 μM FeCl_3_ (human recombinant POLB, 5 min); ≥250 μM FeCl_3_ (rodent brain nuclear extract, 5 min)	[51]
	Zn ↑ (activity ↓)	≥10 μM ZnCl_2_ (human recombinant POLB, 5 min); ≥50 μM ZnCl_2_ (rodent brain nuclear extract, 5 min)	
XRCC1	Cu ↑ (expression ↑)	350 μM CuSO_4_ (SH-SY5Y cells, 24 h)	[92]
DNA damage response & maintenance of genomic stability			
ATM	Mn ↑ (activity ↑)	50 μM MnCl_2_ (mouse striatal cells, 24 h)	[125]
p53	Cu ↑ (activity ↑)	100 μM CuSO_4_ (human NTERA-2-N neurons, 18 h)	[100]
	Mn ↑ (activity ↑)	≥300 μM MnCl_2_ (PC12 cells, 24 h) 50 μM MnCl_2_ (mouse striatal cells, 24 h)	[125,126]
	Zn ↓ (gene expression ↑, binding ↓)	(C6 rat glioma cells, grown in media rendered Zn-deficient)	[17]
	Se ↑ (protein expression ↑, gene expression ↑)	0.5 mM Na_2_SeO_3_ (Primary murine cortical neurons, 2–3 h)	[181]
PARP1/PARylation	Fe ↑ (activity ↑)	5000 mg/L ferric ammonium citrate (C57BL/6J, 3 month)	[44]
	Cu ↑ (expression ↓)	350 μM CuSO_4_ (SH-SY5Y cells, 24 h)	[92]
	Mn ↑ (PARylation ↑)	1 μM MnCl_2_ (cultured human astrocytes, 2 h)	[111]
	Zn ↑ (activity ↑)	400 μM ZnCl_2_ (mixed murine cortical cell culture, 15 min)	[156]
	Se ↑ (activity ↑)	1 μM Se (human glioblastoma cells, 10 h)	[18]

This review aims to comprehensively summarise studies on the impact of excessive and deficient conditions of the TEs Fe, Cu, Mn, Zn, and Se, and the respective consequences on neuronal genomic stability and its maintenance. A special focus is set on the TE-associated generation of oxidative stress and the thereby initiated response of the neuronal genomic stability maintaining machinery.

**Fig. 2:**
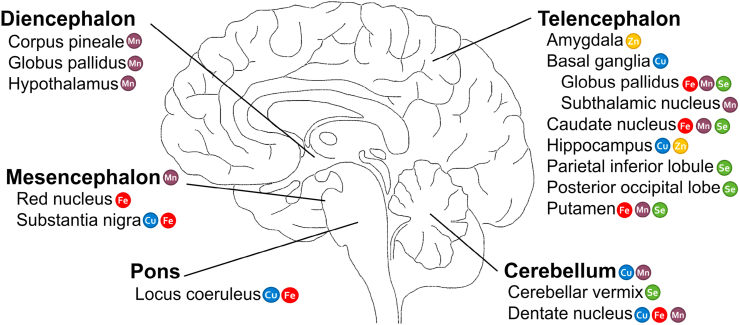
**Schematic overview of brain regions and distribution of Fe, Cu, Mn, Zn, and Se highlighting areas of TE enrichments under physiological conditions.** Illustrated is a schematic longitudinal section of the brain with its five main brain regions. Additionally shown are the respective subsections for each main brain region with their particular TE enrichment. A detailed summary of the different TE brain concentrations is reviewed in Grochowski et al. [31,105].

For this review, a systematic literature search was conducted via PudMed as well as Web of Knowledge. The selection criteria of included literature are listed in Supplementary data Table 1. In general, relevant studies are too diverse in terms of model organisms, study design, and applied methodology to attempt a meta-analysis. The effects of altered TE homeostasis on neurogenesis, as well as on neural progenitor stem cells, occurring in mammalian adult brain alongside with non-self-replenishing post-mitotic neuronal cells, are not covered in this review. A schematical overview of particular TE enrichments in different brain regions under physiological conditions is illustrated in Fig. 2, whereas a detailed summary of the different TE brain concentrations is reviewed in Grochowski et al. [31].

## 2 Iron

In the brain, Fe concentrations are highest among the essential TEs. Particular enrichments have been observed in globus pallidus, substantia nigra, putamen, caudate nucleus, red nucleus, dentate nucleus, and locus coeruleus (Fig. 2) [31,32]. Fe fulfils vital functions in cellular respiration, oxygen transport, myelin synthesis, cellular metabolism, synthesis and repair of DNA, as well as neurotransmitter synthesis and metabolism [[33], [34], [35], [36]]. Most commonly, Fe occurs as divalent ferrous Fe (Fe(II)) and trivalent ferric Fe (Fe(III)) [33,37]. The electron transfer capacity of Fe is fundamental for its biological functions, for example, in enzyme-bound form in mitochondrial respiration and oxygen transport. Yet, at the same time free Fe facilitates the formation of ROS and can cause severe oxidative stress. Fe overload concomitant with an increase in free, highly reactive Fe can result from both exogenous exposure towards Fe and endogenous origin such as disease or Fe dyshomeostasis. Therefore, a tight cooperation of enzymes and transporters involved in Fe homeostasis is of particular importance (reviewed in Ref. [38]).

There is evidence that Fe is involved in oxidative stress-mediated neuronal cell death from a post-mortem study on patients who suffered from neurodegenerative diseases. Age-matched patients who passed away without having shown clinical or pathological symptoms of neurodegeneration were chosen as controls. An increase in total Fe in the affected brain area of neurodegeneration patients was developed in patients with progressive supranuclear palsy (PSP) (globus pallidus of PSP patients 257 ± 19 ng/mg Fe (control 183 ± 22 ng/mg Fe); substantia nigra of PSP patients 301 ± 26 ng/mg Fe (control 188 ± 22 ng/mg Fe)). Additionally, increased concentrations of labile, non-ferritin Fe have been detected in PD and AD (substantia nigra PD patients 534 ± 71 ng/mg H-rich ferritin (control 375 ± 38 ng/mg H-rich ferritin); hippocampus AD patients 29 ± 5 ng/mg L-rich ferritin (control 9 ± 2 ng/mg L-rich ferritin)). These were small, but supposed to be sufficient to trigger Fenton reactions [39]. Another case study on a patient who suffered from hereditary ferritinopathy (HF) revealed consequences of Fe overload for the human brain. In HF, ferritin is structurally and functionally impaired, resulting in Fe dyshomeostasis and brain Fe accumulation [40]. In the HF patient's brain, dramatic cellular loss was observed along with markedly increased brain Fe (putaminal Fe of HF patient 97 μg/g Fe (Huntington's disease patient 2.6 μg/g Fe)) [41]. Primarily in the posterior putamen and cerebellum, neuronal and glial apoptotic cells were observed. Putaminal neurons and glia showed morphological characteristics of lipid peroxidation and abnormal protein nitration, evidently resulting from oxidative stress caused by excessive Fe. Further, swollen to vacuolated nuclei containing ferritin and Fe were observed in these cells, concomitant with severe DNA breakage. Due to immunoreactivity for both p53 and activated caspase-3, a p53-mediated apoptotic pathway was suggested for these cells. Fe dyshomeostasis presumably also deleteriously affected the respiratory chain, indicated by abnormalities in mitochondrial function [41,42].

Accordingly, studies in rodents have shown an increase in oxidative stress in the brain after acute (single dose of 500 mg/kg Fe-dextran) (≙ 8.5 fold increased total Fe brain content compared to control), *intraperitoneal* [4]; 100 μL whole blood or 30 μL FeCl_3_, *intracranial* [43], chronic (3 months, 5000 mg/L ferric ammonium citrate (FAC) via drinking water [44]; 42 days, 1000 mg/L FeCl_2_ (≙ 400% increased Fe cortex level (35 ± 5 μg Fe/g brain)) via drinking water [45], and sub-chronic Fe overload (10 days, 6 doses of 50 mg/kg Fe-dextran, *intraperitonea*l [46]; 4 weeks, 5 days/week, 50 mg/kg Fe-dextran, *intraperitoneal* [47]). In rat brains, an increase in 8-oxo-7,8-dihydro-2′-deoxyguanosine (8-oxodG) and AP sites was observed by immunohistochemistry following both experimental intracerebral haemorrhage via injection of whole blood and direct injection of soluble Fe (100 μL whole blood or 30 μL FeCl_3_, *intracranial*) [43]. In response to Fe-induced oxidative stress, activation of nuclear factor κB (NF-κB) and of the antioxidant enzymes CAT, superoxide dismutase (SOD) 1, and GPX4 have been observed [4,44,47]. Additionally, in chronic Fe overload (42 days, 1000 mg/L FeCl_2_ (≙ 400% increased Fe cortex level (35 ± 5 μg Fe/g brain) via drinking water) in rodents a marked decrease in GSH and reduced/oxidized GSH ratio was observed [45], which is supposedly dependent on the overall antioxidant capacity and the actual severity of Fe overload. To protect cellular components such as proteins, lipids, and DNA from oxidative damage, in acute Fe overload, the antioxidant system was presumably initiated by increased NF-κB binding (8 h post treatment) which in turn resulted in increased CAT activity (21 h post treatment) [4]. In contrast to acute Fe overload, no changes in NF-κB DNA binding activity were observed in response to sub-chronic Fe overload although CAT and SOD activities were increased [46]. The authors hypothesise that these observations may be caused by activation of nuclear factor erythroid 2-like 2 (Nrf2) activation, which is a redox-sensitive transcription factor alike NF-κB, that is involved in regulation of SOD and CAT, and was shown to be activated upon sub-chronic Fe-administration (every second day for 10 days, 50 mg/kg Fe-dextran, *intraperitoneal*) [48].

With increasing concentrations of Fe administration, the observed adverse effects induced in the brain by Fe-derived reactive species increase. Fe(II) and Fe(III) that is not directly administered into the brain or released into the brain due to pathological conditions, needs to cross the blood-brain barrier (BBB) to impact on neurons. In a state of Fe overload, capacity of transferrin to bind circulating Fe may be exceeded, resulting in high concentrations of non-transferrin-bound Fe facing the BBB. In addition to transferrin receptor-meditated uptake of transferrin-bound Fe(III), this free Fe(II) is suggested to be able to pass the BBB via divalent metal transporter 1 or by binding secreted ferritin, a molecule capable of transporting up to 4,500 Fe atoms, and ferritin receptor-mediated uptake [49]. Thus, high circulating Fe concentrations are likely to cause brain Fe overload concomitant with increased ROS production. If reactive species prevail in a state of Fe dyshomeostasis, genomic integrity may be compromised. A drastic decrease in mitochondrial and nuclear DNA integrity determined by quantitative PCR after acute Fe overload (single dose of 500 mg/kg Fe-dextran (≙ 7.7 fold increased total brain Fe content (5.4 ± 1.5 pmol/mg Fe) after 6 h), *intraperitoneal*) has been reported in rat brain, by 65% and 41%, respectively. However, after sub-chronic Fe overload (6 doses of 50 mg/kg Fe-dextran (≙ 7.6 fold increased total brain Fe content (6.5 ± 0.25 pmol/mg Fe) after 6 doses), *intraperitoneal*) mitochondrial DNA integrity decreased by only 25%, whereas nuclear DNA integrity was not affected [50]. These results substantiate the hypothesis that in sub-chronic Fe overload, cellular protective mechanisms bear the capacity to safeguard genomic integrity by restoring Fe homeostasis and activating the antioxidant systems, while in acute Fe overload the capacity is exceeded.

Next to DNA integrity, Fe directly impacts on BER, the fundamental protective DNA repair pathway in the brain. BER key enzymes have been shown to be affected by Fe. Applying cerebral nuclear extract from rat brains in a DNA repair assay with recombinant DNA repair enzymes, AP site incision activity (10–250 μM FeCl_3_, effect observed ≥ 10 μM) and ligation of DNA (10–250 μM FeCl_3_, effect observed ≥ 10 μM) were dose-dependently inhibited and gap-filling synthesis by polymerase ß (POLβ) (10–250 μM FeCl_3_, effect observed ≥ 10 μM) was blocked [51]. In murine brains, in response to chronic Fe exposure (3 months, 5000 mg/L FAC via drinking water), expression of DNA repair proteins e.g., mammalian mutY homologous DNA glycosylase (MUTYH), 8-oxodG DNA glycosylase (OGG1), and nudix hydrolase was significantly decreased as determined by Western Blot analysis [44]. Low Fe(II/III) concentrations (0.1–10 μM FeSO_4_/FeCl_3_, effect observed ≥ 10 μM) strongly inhibited total repair of a 5-hydroxyuracil-containing oligonucleotide initialised by activity of human recombinant nei like DNA glycosylase (NEIL) 1 and 2. Additionally, applying human recombinant proteins in affinity co-elution analysis, Fe(II) (10 μM) was shown to severely inhibit the interaction of NEIL1 with the downstream BER enzymes POLβ and flap structure-specific endonuclease 1 (FEN1). In undifferentiated human neuroblastoma SH-SY5Y cells, Fe (100 μM FeSO_4_) lowered 5-hydroxyuracil excision from a bubble substrate (NEIL1/2 activity) and AP lyase activity by 50–60% [52]. Accordingly, alkaline comet assay analysis revealed a significant reduction in progression of repair of oxidatively damaged genomic DNA by Fe(II/III) (2–4 h, 50 μM FeSO_4_/FeCl_3_, effect observed ≥ 4 h), in cultured primary mouse neurons [51].

Disturbance of the repair pathway for oxidative damage is especially worrisome as Fe overload has been verified to increase ROS production in various model systems such as mouse hippocampal cells (24–72 h, 150 μM FAC, effect observed ≥ 24 h) [53], dopaminergic (DAergic) human neuroblastoma SK-N-SH cells (100–250 μM FeSO_4_, effect observed ≥ 100 μM) [54], primary rat microglial cells, and BV-2 microglial cells (100 μM FeSO_4_) [55]. The latter study included a co-culture experiment between primary neurons and activated BV-2 microglial cells. Only the microglial cells were exposed to Fe, yet after indirect co-culture the elevated ROS levels resulted in increased neuronal death showing the range of destructive potential of ROS formation in the brain. According to the human situation, secondary effects resulting from Fe-induced ROS production have been reported in the investigated model systems. These include an increase in lipid peroxidation (1 h, 200 μM FeSO_4_) [56], mitochondrial fragmentation, and endoplasmatic reticulum stress (24–72 h, 150 μM FAC, effect observed ≥ 24/48 h) [53]. In addition, an increase in metallothionein and GSH have been observed in response to increased Fe exposure in SK-N-SH cells (overnight up to 24 h, 100–1000 μM FeSO_4_, effect observed ≥1000 μM) [54], which could be interpreted as cellular attempt to counteract Fe-induced oxidative stress and safeguard genomic integrity. However, in the latter study in consequence of Fe-induced (30 min, 100 μM FeSO_4_) ROS production, NF-κB-mediated neuronal apoptotic cell death was observed, thus suggesting that the antioxidative defence was exceeded under study conditions [54].

Compromised BER in response to chronic high dietary Fe may result in high DNA damage levels, potentially resulting in neuronal loss. Chronic exposure of high dietary Fe in mice (3 months, 5000 mg/L FAC via drinking water [44]; 4 weeks, 5 days/week, 50 mg/kg Fe-dextran, *intraperitoneal* [46]) resulted in structural and functional neuron loss in the cortex and hippocampus, and in rats after acute Fe overload (single dose of 500 mg/kg Fe-dextran, *intraperitoneal* [43], single *intrahippocampal* dose of 10 μL bovine hemoglobin (150 mg/mL) [57] or FeCl_2_ (1 mM) [58] or 6 doses of 50 mg/kg Fe-dextran every second day, *intraperitoneal* [4,50]). Neuronal loss was described to occur via apoptosis in a B-cell lymphoma 2 (BCL2) associated X protein, PARP1, and apoptosis-inducing factor-dependent manner in mouse brains [44], along with an activation of PARP observed in rat brains 24 h after intracerebral hemoglobin infusion (30 μL, 300 mg/mL bovine hemoglobin) [58]. In line with this, in rats exposed to Fe via subarachnoid haemorrhage (SAH) (101 ± 16 μg Fe/g brain tissue (control animals 43 ± 10 μg Fe/g brain tissue), 3 days after SAH), numerous apoptotic and 8-oxodG-positive cells were observed [59], corroborating the assumption of Fe-induced oxidative stress causing apoptosis.

Increased ROS production and oxidative damage were also shown in induced pluripotent stem cell (iPSC)-derived neurons from HF patients. HF-iPSC neurons are characterised by increased cytosolic free Fe and altered Fe homeostasis, and hence resemble the condition of HF. In addition to increased oxidative stress, HF-iPSC neurons showed by 50% reduced cell viability, increased DNA damage, and a drastic decrease in DNA stability maintaining nuclear receptor coactivator 4. Further, the obtained results indicate activation of the p53 signalling pathway [60]. p53 in turn is capable of activating Fe-dependent ferroptosis [61]. Evidently, in response to high cytosolic free Fe, human HF-iPSC neurons show an increased tendency to die by ferroptosis [60]. Ferroptosis is a regulated cell death pathway, initiated by iron-dependent lipid peroxidation, a process that can be antagonized by the selenoprotein GPX4 [62]. In contrast, in SK-N-SH cells caspase-3 activation in response to Fe overload (overnight exposure, 100 μM FeSO_4_) was observed, in line with the human HF situation, concomitant with mitochondrial damage, nuclear DNA condensation, and fragmentation following NF-κB activation and BCL2 inhibition [54]. Primary cultures of rat cerebellar granule cells, next to a severe decrease in cell viability, showed typical morphological signs of apoptosis as evident by transmission electron microscopy and “ladder pattern” DNA fragmentation occurring during electrophoresis following 1 h treatment with 200 μM FeSO_4_. Especially intra-nucleosomal cleavage of DNA as observed, is a hallmark of apoptosis. In addition, caspase-3-like activity increased and p53 expression was upregulated 2.1-fold [56]. Accordingly, apoptosis was observed in SH-SY5Y cells exposed to 1–5 mM Fe (FAC, effect observed ≥ 25 mM) for 48 h, but determined by the use of an Annexin V staining-based commercially available kit. Further, activated caspase-3 and cleaved PARP were significantly upregulated, which facilitated apoptotic progression [63]. These results are in agreement with the well-known p53-mediated apoptotic pathway for neuronal cell death with caspase-3 activation as key determinant [64]. Thus, this contradicts the hypothesis of an initiation of ferroptosis. Yet, as ferroptosis is mainly induced by Fe accumulation concomitant with lipid peroxidation [65], the cell death pathway might be determined by the type of biomolecule which is the main target of oxidative damage. This question remains to be answered as the mechanisms of Fe overload in ferroptosis and the pathway choice between apoptosis and ferroptosis are not fully elucidated yet [66].

In summary, Fe overload, irrespective of an exogenous or endogenous origin, severely affects neuronal genomic stability. Massive ROS production in response to brain Fe overload starts the cascade of increased DNA damage, whose repair is impaired by Fe, resulting in apoptotic and ferroptotic neuronal death. Eventually, structural and functional loss of brain tissue causes a severe pathological outcome for patients suffering from brain Fe overload.

## 3 Copper

Cu is the third abundant TE in the brain after Fe and Zn, with highest levels in the substantia nigra, locus coeruleus (both containing catecholaminergic cells), dentate nucleus, basal ganglia, hippocampus, and cerebellum (Fig. 2) [31]. It is essential for fundamental physiological processes in the brain, being involved in cellular respiration, antioxidant system, energy metabolism, Fe metabolism, neuropeptide and neurotransmitter synthesis [67]. To allow for fulfilling its diverse functions and at the same time for preventing ROS formation by its redox activity (Cu(I) and Cu(II)), Cu homeostasis needs to be maintained and regulated [67,68]. Systemic as well as local Cu dyshomeostasis has been associated with certain neurological diseases, such as WD, AD, and PD [[69], [70], [71]].

Microarray studies with RNA samples from brains of AD patients revealed reduced mRNA expression of the Cu-dependent enzymes SOD1 and antioxidant protein 1 which was associated with a reduced Cu concentration in the brain. This indicates that low Cu levels could induce oxidative stress via downregulation of neuronal antioxidant systems [72,73]. Furthermore, in a human case report, reduced serum Cu levels (0.09 μg/mL Cu (normal 0.75–1.45 μg/mL)) due to malabsorption were associated with neuropathological findings. These neurological impairments could be reversed following Cu supplementation (10 months, 3 mg/day Cu(II)), pointing out the importance of an adequate Cu homeostasis for physiological brain function [74]. Besides deficiency, excess Cu levels in the brain, as observed in WD, have been associated with impaired neuronal function as well [22]. Compared to healthy individuals, serum Cu levels of AD patients were 2.2 fold elevated and brains of diseased patients showed increased levels of Cu, particularly enriched in amyloid plaques (0.4 mM Cu) [75,76]. There are indications for reduced DNA repair capacity in brains of AD patients [77,78]. Several double-strand break repair proteins, which are responsible for the cellular response to DNA damage, were downregulated in brain samples from AD patients (total Cu brain content ≤ 400 μM) compared to age-matched non-AD controls (total Cu brain content = 70 μM). Furthermore, AD patients showed increased levels of oxidative DNA damage. Compared to healthy individuals, AD patients have been reported to have twice the level of 8-oxodG in the cerebrospinal fluid [79].

Consistent with human results, studies in rodents provided a link between an overload of Cu and neurological dysfunction. Excessive Cu levels in brain were again associated with increased oxidative stress [10,80]. In this context, Musacco-Sebio et al. observed an increase in phospholipid peroxidation and protein oxidation likely mediated via free radical generation of hydroxyl radicals in rats receiving increasing dosages of Cu (16 h, 0–30 mg/kg CuSO_4_, *intraperitoneal,* total Cu brain content 34 ± 2 μg Cu/g brain (control 3.6 ± 0.8 μg Cu/g brain)) [10]. Moreover, an induction of DNA damage in connection with elevated brain Cu levels was observed in several animal models for WD [81,82].

*In vitro* studies in human SH-SY5Y neuroblastoma cells confirmed an increase in oxidative stress following exposure to Cu determined via flow cytometry (24 h, 50–300 μM CuSO_4_, effect observed ≥ 150 μM (≙ cellular 50 ng Cu/mg protein)) [83]. For Cu-induced oxidative stress, additional mechanisms are supposed to play a role besides Fenton-like mechanisms. In brain, neurotransmitters such as dopamine might be involved in the redox-cycling process. Cu promotes the oxidation of dopamine via its semiquinone radical or quinone intermediate resulting in formation of diverse reactive species and eventually DNA damage [[84], [85], [86], [87]]. Moreover, other proteins might be involved in ROS formation such as amyloid-β complexes (plaques associated with AD). In support of this, a study with human tissue slices of affected brains reported increased oxidative damage at amyloid plaque regions [88]. Most likely, the amyloid precursor protein contributes to Cu-mediated ROS formation by providing Cu-binding properties and thereby enabling redox reactions via its reductase activity [87,[89], [90], [91]].

So far, there are no clear indications for any direct genotoxic potential of Cu. The observed increased DNA lesions in response to Cu are rather supposed to occur via inhibition of the repair mechanism [22], studies focussing on this relationship are limited. Cu has been shown to interact with BER at different levels. Induction of (oxidative) DNA damage and a concomitant impairment of BER repair mechanisms was studied in the human neuroblastoma SH-SY5Y cell line after Cu incubation (24 h, 350 μM CuSO_4_). OGG1 mRNA expression levels were significantly increased, while enzyme activity of this key BER enzyme, mainly responsible for excision of 8-oxodG, was not affected. Furthermore, significant changes in gene expression could be determined for X-ray repair cross-complementing 1 (XRCC1) (upregulation) and damage sensor PARP1 (downregulation) [92]. Additionally, Cu (24 h, twice 0–50 μM CuCl_2_, effect observed ≥ 10 μM) shows inhibitory effects on NEIL activity already at micromolar levels in human neuroblastoma SH-SY5Y cells. Studies with the isolated proteins revealed that Cu binds to NEIL1 and NEIL2 and thus affects their base excision, AP lyase, and strand excision activity [52]. However, it appears that the mode of action of Cu differs between the glycosylases. In case of NEIL1, inhibition of activity is likely mediated via oxidation of cysteine residues of the protein leading to conformational changes and consequently impaired enzyme function. The oxidation of cysteine in NEIL1 was found to be reversible. In fact, the use of Cu chelators combined with a reductant lead to almost complete reactivation of the isolated enzyme. Protective effects by chelating agents against Cu-mediated inhibition of NEIL1 could be also confirmed in human neuroblastoma SH-SY5Y cells. In contrast to NEIL1, conformational changes by Cu could not be confirmed in isolated NEIL2. As NEIL2, but not NEIL1, contains a Zn finger motif, Cu could replace Zn from this specific structure and consequently affect its function [52,93]. This indicates that the observed inhibitory effects of Cu are highly specific. Consequently, direct binding of Cu to the protein is discussed as underlying mechanism for the inhibitory effect on DNA repair, rather than interaction with the DNA. Additional studies highlight the effect of Cu on other downstream proteins of the BER pathways, including POLβ, polynucleotide kinase 3′-phosphatase (PNKP), and PARP1 in recombinant or isolated proteins [79,[94], [95], [96]].

Increased Cu levels in the brain have been shown to cause neuronal damage leading to cell death. Cu is reported to promote apoptosis as observed via chromatin condensation and caspase activation in neurons [[97], [98], [99]]. However, underlying mechanisms on the molecular level are not completely clear. Studies in cultured human NTERA-2-N neurons showed that Cu-induced neuronal apoptosis (18 h, 50–200 μM CuSO_4_, effect observed ≥ 100 μM (≙ 75 μg Cu/mg protein)) is dependent on the induction and nuclear translocation of the transcription factor p53 [100]. Accordingly, downstream target genes, such as p21 were affected by Cu (6–18 h, 100 μM CuSO_4_, effect observed ≥ 18 h; 24 h, 50–75 μM Cu(II)) [97,99,101,102]. Additionally, p53-independent mechanisms are discussed to be involved. Cu-treatment (6–18 h, 100 μM CuSO_4_, effect observed ≥ 18 h; 24–48 h, 0–100 μM Cu-glycine_2_, effect observed ≥ 100 μM, 24 h) of neuroblastoma cells revealed changes in gene expression level of BCL2, c-FOS, and GPX [99,103]. Both mechanisms would result in Cu-induced apoptosis, determining the neuronal fate.

Overall, disrupted Cu homeostasis, particularly in the brain, can result in pathological conditions associated with neurodegenerative diseases such as AD. Potential modes of action appear to be among others the induction of oxidative stress and resulting DNA damage. Moreover, Cu has been shown to disrupt important steps of BER. Consequently, imbalances in Cu levels in the brain likely affect the brain, leading to neuronal damage or cell death.

## 4 Manganese

Mn participates in several biological processes as it is incorporated into various metalloproteins, such as phosphoenolpyruvate decarboxylase, glutamine synthetase, arginase, pyruvate carboxylase, and Mn-SOD enzymes. As those enzymes are involved in regulating the cellular redox status, mitochondrial function, and neurotransmitter synthesis, the Mn supply of the brain is of central importance for brain and cognitive functioning [104], highlighting the importance of a well-regulated homeostatic system (reviewed in Ref. [105]). While Mn deficient conditions are exceptionally rarely observed [3], consequences of excessive and prolonged Mn exposure exceeding the homeostatic range can affect both motor and higher order cognitive functions. The resulting devastating neurological impairment is termed “manganism” and shares characteristic clinical features comparable to those of PD [104]. Although many hypotheses are discussed in literature, the underlying cellular and molecular mechanisms by which Mn induces neurodegeneration are still under investigation. Yet, brain cells are particularly sensitive to Mn and Mn levels in the human brain have been found to be highest in the putamen, caudate nucleus, and globus pallidus (Fig. 2). Additionally, human Mn brain levels correlate positively with age and significantly increased Mn levels were observed in PD diseased brain [106].

Nowadays, data on the impact of Mn on the human brain are very scarce and Mn-induced DNA damage or effects on the DDR are not directly addressed. However, in a study with residents from Mexico City, chronically exposed to fine particulate matter above the annual standard, the brain region-specific effects of metals on key neuroinflammatory markers and DDR were investigated. Frontal cortex Mn concentrations (Mexico City subjects 1026 ± 47 μg/g dry tissue (control 689 ± 119 μg/g dry tissue)) and Se (Mexico City subjects 1111 ± 148 μg/g dry tissue (control 641 ± 312 μg/g dry tissue)) increased with age in exposed subjects and especially for Mn a correlation with neuroinflammation indicated by the upregulation of frontal cortex interleukin 1 beta and cyclooxygenase 2 was observed. In contrast, no significant difference could be determined in DDR gene expression between exposed and control study participants [107]. Moreover, in chronic Mn (10 months, 3.3–5.0 mg Mn/kg, *intravenous*) exposed non-human primates (*Cynomolgus macaques*), gene expression changes indicated an impact of Mn on cell cycle regulation, DNA repair, apoptosis, ubiquitin-proteasome system, protein folding, cholesterol homeostasis, axonal/vesicular transport, and inflammation. p53 was suggested to be the central key player for the Mn-induced neurodegeneration in the frontal cortex [108].

In a rodent study, Bahar et al. exposed rats towards Mn (every 24 h, 8 doses 15 mg/kg MnCl_2,_ *intraperitoneal*), which resulted in Mn-induced oxidative stress in the brain indicated by increased ROS formation and protein carbonyls, and a decrease of Cu/Zn-SOD activity, accompanied by Mn-induced neuroinflammation. Immunohistochemistry of the striatum region revealed a significant increase of the most common oxidative DNA lesion 8-oxodG [109]. Exposing OGG1 knockout mice towards Mn (21 days, 0.005–0.01 mg/L MnCl_2_ via drinking water, effect observed ≥ 0.01 mg/L) sensitises DAergic neurons to Mn-induced toxicity. Dopamine was reduced in the caudate of OGG1 knockout mice. Additionally, the reduction of dopamine in caudate putamen correlated with the accumulation of 8-oxodG in the midbrain in the OGG1 knockout mice indicating that OGG1 function is essential in maintaining neuronal genomic stability [110].

With respect to the different cell types of the brain, bioavailability data reveal that human astrocytes CCF-STTG1 may not be the main targets of Mn neurotoxicity (2 h, 100–500 μM MnCl_2_ (≙ cellular 50–140 μM)) [111]. Neurons are reported to be more susceptible to Mn intoxication possibly due to their long lifespan and high energy demand [112]. Mn neurotoxicity appears to be regulated in brain cells by multiple factors, including oxidative injury, mitochondrial dysfunction, and protein misfolding [111,113,114]. By its redox-activity, Mn can increase the formation of reactive oxygen and nitrogen species (RONS) directly or indirectly by inhibiting complex I-IV activity of the mitochondrial electron transfer chain enzymes [112]. Additionally, current metabolomic analyses show that exposure of human SH-SY5Y neuroblastoma cells to Mn (5–24 h, 0–100 μM MnCl_2_, effect observed ≥ 50 μM, 5 h) causes changes in energy homeostatic systems. The Mn-induced disruption in energy and fatty acid metabolism results in subsequent cell death [115,116]. Regarding the intracellular Mn distribution few studies suggest that the lysosomes, the Golgi apparatus, the endosome, mitochondria as well as the nucleus may be significant pools for intracellular Mn [105]. Thereby, especially the nuclei are currently discussed to serve as the primary pool for intracellular Mn. Mn (24 h, 100 μM MnCl_2_) accumulated mainly in the nuclei of cultured choroidal epithelial and brain endothelium cells and in the nuclei and cytoplasm of cultured DAergic neurons upon exposure while in mitochondrial and microsomal fractions less Mn was detected [117]. Taking excessive RONS production as well as the recent investigations of the intracellular Mn distribution into account, this underlines the importance to have a closer look at genomic stability upon Mn exposure.

In human neuroblastoma SH-SY5Y cells, Mn (24 h, 2–125 μM Mn(II)) induced single-strand breaks (detected by alkaline comet assay, effect observed ≥ 2 μM Mn(II)) as well as oxidative damage to DNA bases (2,6-diamino-4-hydroxy-5-formamidopyrimidine, 8-oxodG, and 5-OH-5-MetHyd lesions, effect observed ≥ 62 μM Mn(II)) detected by mass spectrometry [118]. In the rat-derived neuronal PC-12 cells, Mn exposure (24–72 h, 0.01 μg/mL MnCl_2,_ effect observed ≥ 24 h) resulted in elevated lipid peroxidation without excessive accumulation of oxidative DNA damage as measured by 8-oxodG [119]. However, in a further study using PC-12 cells, Mn (36 h, 200 μM MnCl_2_) increased the 8-oxodG content in the DNA of dopamine treated PC-12 cells [120]. Upon Mn treatment (24 h, 100–400 μM MnCl_2,_ effect observed ≥ 200 μM), primary neurons and astrocytes isolated from mice showed decreased levels of GSH and increased 8-oxodG and carbonyl levels in a dose-dependent manner. Within this study, the observed GSH depletion is a key factor for oxidative damage during Mn exposure [121]. In cultured human astrocytes, Mn (2–48 h, 100–500 μM MnCl_2_) did not significantly induce DNA stand breaks [111]. Studies identifying the interaction of DNA repair genes with Mn in brain cells are so far lacking. A study with recombinant human DNA polymerase indicates an impaired activity of DNA polymerase iota by Mn [122]. Regarding DDR, PARP1 inhibition has been shown to diminish mitochondrial capacity and rate of DNA repair with severe consequences for neuronal cells such as cell death [123]. In human astrocytes, damage-induced poly(ADP-ribosyl)ation (PARylation) was significantly inhibited already at physiologically relevant non-cytotoxic Mn concentrations (2 h, 1 μM MnCl_2_), identifying the DNA damage-related signalling reaction PARylation as highly sensitive to *in vitro* Mn exposure [111]. The underlying mechanisms still need to be clarified as Mn (2–48 h, 0–100 μM MnCl_2_) did not decrease PARP1 gene expression or PARP1 protein level at any time point investigated [111]. Ataxia telangiectasia mutated serine/threonine kinase (ATM) is a Mn-activated enzyme involved in the cellular response to DNA damage and is mutated in Ataxia telangiectasia, a neurodegenerative autosomal recessive disorder with impaired mitochondrial function and deficient mitochondrial DNA repair. ATM has been shown to phosphorylate p53, H2AX, and other targets in response to increased Mn levels, DNA damage, and oxidative stress [124]. In mouse striatal cells, activation of ATM kinase activity was shown to be sensitive to Mn (24 h, 50 μM Mn(II) (≙ cellular 0.5 nm/μg of DNA)) and studying inhibitors confirmed ATM-p53 as an important pathway responding to Mn exposure [125]. A major p53 response to Mn exposure was also identified in mouse striatal cells and PC-12 cells, providing further evidence of the role of p53 in Mn neurotoxicity [125,126]. Additionally, markers like caspase-3 activation as well as PARP cleavage facilitate apoptotic progression of neuronal cell death in PC-12 cells and dopamine producing catecholaminergic cells [126,127].

To conclude, excessive Mn exposure may result in neurological impairment characterised by loss of DAergic neurons. Recent studies indicate a contribution of Mn-induced oxidative stress, genomic instability by means of Mn-induced DNA damage and the impact on DDR to apoptosis-mediated cell death.

## 5 Zinc

In comparison to other organs in the human body, Zn concentrations are highest in brain exceeding e.g. liver and serum concentrations by a factor of 10 [31,128]. Elevated Zn levels are found in hippocampus, amygdala, and dentate gyrus (Fig. 2) [31,129]. 80–90% of neuronal Zn are tightly bound to metal-binding proteins, whereas the remaining fraction is stored in synaptic vesicles of a large sub-population of excitatory neurons [130]. In its divalent form, Zn is a catalytic, structural, or regulatory component of various proteins, which are involved in a multitude of physiological processes. These include neurotransmission, enzymatic activity, gene regulation, as well as structural preservation and stabilization of proteins [[130], [131], [132], [133]]. To fulfil its versatile functions within neurons and to maintain physiological brain function, intracellular Zn homeostasis has to be strictly regulated (reviewed in Refs. [128,134]). Both Zn-deficient as well as - excessive conditions are linked to increased oxidative stress and pathophysiological neuronal changes such as ageing-associated diseases [19,135,136]. However, human data on the impact of Zn on oxidative stress as well as relating DDR and DNA repair are not available.

Feeding studies in rodents emphasise the essentiality of an adequate Zn supply for physiological brain function with regard to the maintenance of the brain's redox balance [[137], [138], [139], [140]]. Mice, which obtained a Zn deficient diet (5 weeks, 1–2 mg/kg Zn), revealed increased lipid peroxidation and DNA fragmentation, GSH depletion, and increased ROS production in brain tissue. These effects could be circumvented by a Zn supplementation (4 days, 13 mg/kg Zn), whereby Zn l-methionine was most efficient [137]. Comparable results were gained in a Zn-deficient intervention study (12 weeks, 0.3 mg/kg Zn) in rats. Lee et al. showed besides altered GSH and ROS levels compared to the control group (12 weeks, 60 mg/kg Zn (≙ cortex Zn content 14.8 ± 3.1 ppm (control 14.8 ± 6.4 ppm), hippocampus Zn content 21.4 ± 6.5 ppm (control 20.4 ± 9.1 ppm)), decreases in CAT, GSH reductase, and GPX activities in cortex, next to decreased SOD activity in the hippocampus [138].

Moreover, several *in vitro* studies demonstrated the link between imbalances in Zn homeostasis and increased oxidative stress levels [17,141]. The role of Zn in increasing oxidative damage may be related to (i) the modulation of redox homeostasis by Zn, (ii) changes in expression of Zn-binding metallothioneins or (iii) an altered mitochondrial function [132,142]. In addition, a mechanism including the N-methyl-d-aspartate receptor (NMDAR) was discussed. Differentiated PC-12 cells cultured in Zn-deficient medium (1.5 μM Zn) showed an increase in RONS by activation of NMDAR leading to calcium (Ca) influx and to a Ca-mediated activation of protein kinase C/nicotinamide adenine dinucleotide phosphate oxidase [143]. Moreover, besides increased oxidative stress, several studies in neuronal cell lines revealed correlations between deficient Zn condition and impaired DDR, and DNA repair pathways [17,141]. Ho et al. showed increased oxidative stress and impairment of several DDR steps in the rat glioma cell line C6, grown in media rendered Zn-deficient by chelation with Chelex-100 (5 days, Zn deficient media (≙ cellular Zn content 0.035 μg/million cells (control 0.05 μg/million cells))). Alongside with an increased expression of p53 and apurinic endonuclease 1 (APE1), the DNA-binding ability of the transcription factors p53, NF-κB, and activator protein 1 was decreased, highlighting the potential detrimental effect of Zn deficiency on DNA damage response. Moreover, under Zn deficient conditions (24 h, 1.5 μM Zn (≙ cellular 4.1 ± 0.3 nmol/mg protein)) in human neuroblastoma IMR-32 cells, NF-κB nuclear translocation was impaired, inhibiting the expression of its target genes and hence affecting cell survival [141]. Activation of p53 is discussed to play a central role in apoptosis triggered by Zn deficiency. p53 is a key modulator of Zn chelator N,N,N′,N'-Tetrakis(2-pyridylmethyl)ethylenediamine (TPEN)-induced neuronal apoptosis, mediating the expression of p53 upregulated modulator of apoptosis and NOXA and caspase-11-mediated activation of caspase-3 in a primary mouse cortical cell culture [144]. However, results using TPEN to decrease cellular Zn should be interpreted carefully due to its binding affinity to other transition metals and its toxic effects [145]. Substantial evidence links Zn deficiency to neuronal cell death via the intrinsic apoptosis pathway [146]. In Zn-deficient medium (1.5 μM Zn), human neuroblastoma IMR-32 cells and primary cultures of rat cortical neurons showed a decrease in cell viability combined with caspase-3 activation as well as alterations in cell signalling, regulating the expression of pro-survival or pro-apoptotic genes contributing to Zn deficiency-induced apoptosis [146].

Concurrently, several components of the antioxidant system were negatively affected by Zn deficiency [147,148]. Among others, Omata et al. observed an impaired neuronal cell capacity to upregulate components of GSH synthesis pathway as a protective response to oxidative stress. Coincident with a decreased GSH level, a decreased expression on mRNA and protein level of the catalytic and modulatory subunit glutamate cysteine ligase (GCL), a central enzyme in GSH synthesis, was observed. Among others the activation of the GCL subunit expression regulating transcription factor nuclear factor erythroid 2-like 2 activation was impaired in neuronal IMR-32 cells grown in Zn-deficient medium (1.5 μM Zn) [148]. These findings emphasise that an impaired capacity to regulate GSH metabolism under Zn-deficient conditions increases the vulnerability of neurons to accumulate oxidative damage.

Not only Zn deficiency, but also Zn excess impacts on neuronal DNA repair. Li et al. identified a delayed repair of oxidatively damaged genomic DNA of cultured primary mouse neurons in the presence of Zn (4 h, 50 μM ZnCl_2_) measured by alkaline comet assay. Furthermore, impact of Zn on key BER steps was examined in a DNA repair assay with recombinant DNA repair enzymes and cerebral nuclear extract from rat brains. Zn showed an inhibitory effect on several BER enzymes such as FEN1 (50–250 μM ZnCl_2,_ effect observed ≥ 250 μM) and POLβ (50–250 μM ZnCl_2,_ effect observed ≥ 50 μM), albeit impact on the final ligation step was most pronounced even at low Zn concentrations (10–250 μM ZnCl_2,_ effect observed ≥ 10 μM) [51].

Regarding acute Zn neurotoxicity, free Zn(II) translocated from presynaptic vesicles plays a unique role in the pathophysiology of several neurodegenerative diseases such as AD [[149], [150], [151]]. It can trigger ROS production through a number of signalling processes, resulting in a positive feedback loop and numerous adverse effects [152]. In cultured murine neocortical cells, exposure to high Zn concentrations (15 min, 1000 μM ZnCl_2_) resulted in neuronal death visualised by microscopy [153]. Furthermore, murine cortical neurons exposed to high Zn concentrations (24 h, 35 μM Zn) exhibited signs of necrosis such as body swelling and destruction of intracellular organelles, while higher concentrations (24 h, 40 μM Zn) resulted in complete neuronal cell death [154]. In addition, there is evidence that Zn-induced necrosis is linked to PARP activation [155]. A mixed murine cortical cell culture, containing both neurons and astrocytes, exposed to Zn overload (15 min, 400 μM ZnCl_2_) showed DNA fragmentation and increased PARylation, concomitant with decreased levels of nicotinamide adenine dinucleotide and ATP, eventually resulting in cell death [156].

In conclusion, imbalances in Zn homeostasis enhance vulnerability of neuronal cells to oxidative DNA damage by impairment of antioxidant systems. Furthermore, recent studies highlight the influence of excessive Zn inhibiting key BER enzymes leading to impaired DDR and DNA repair pathways consequently resulting in an affected neuronal genome maintenance.

## 6 Selenium

Se is a very important TE for maintaining brain function. Within the whole organism, it is preferentially transported to the brain at the expense of other tissues to ensure sufficient Se concentrations even under conditions of nutritional deficiency. However, in comparison to other organs such as liver or kidney, the total brain Se content is relatively low [157,158]. Se as selenocysteine becomes incorporated into selenoproteins. In humans, 25 genes encode for selenoproteins, and most of them have been described to be important for redox regulation, especially the GPX and thioredoxin reductases (TXNRD). Selenoprotein P (SELENOP) is essential for transporting Se from the liver to peripheral organs such as the brain by binding to the apolipoprotein E receptor 2 (APOER2) [159]. Different syndromes of impaired selenoprotein expression in humans are accompanied by neurological symptoms indicating that selenoproteins are essential for proper brain function also in humans [160]. A knockout of SELENOP or APOER2 in mice markedly reduces the Se content in brain, local selenoprotein expression, and results in poor motor coordination, seizures, and ataxia [[161], [162], [163]]. Under Se deficiency (8 weeks, Torula yeast-based diet; 4 weeks, 0.025 mg Se/kg (≙ Se brain content 125 ng/g tissue)), murine brain Se concentrations were only marginally affected in comparison to other organs [157,164]. Lower Se concentrations were detected in cortex, midbrain, brainstem, and cerebellum but not in the hippocampus, which was only affected by loss of SELENOP [157]. Besides the liver, SELENOP is also locally expressed in the brain. The liver-specific deletion of SELENOP in mice revealed that hepatic SELENOP is the main transporter of Se but local SELENOP expression is required to maintain the Se content in brain during dietary Se restriction (12 weeks, 0.06 mg Se/kg) [165]. The most susceptible cells to loss of SELENOP appear to be γ-aminobutyric acidergic parvalbumin-positive interneurons which is most probably caused by an increase in oxidative stress upon loss of SELENOP and additional selenoproteins [166]. Essential selenoproteins in the brain include also GPX4 and TXNRD1. Neuron-specific GPX4 knockout mice suffer from neurodegeneration and loss of parvalbumin-positive interneurons of the cortex. Additional neuronal subpopulations that depend on GPX4 expression include hippocampal neurons, glutamatergic neurons, cerebellar Purkinje cells, and motoneurons, while neuronal subpopulations of the hypothalamus and dopaminergic neurons are resistant to loss of GPX4 (overview in Ref. [62]). Transgenic mouse models with loss of TXNRD1 revealed that this selenoprotein is important in radial glia cells and thus for long term maintenance of neurons (overview in Ref. [167]).

As a consequence of reduced selenoprotein expression, oxidative stress occurs in the brain [168]. For other organs it has been well described that low Se concentrations and accelerated levels of oxidative stress result in genomic instability [169]. However, studies showing this relationship are rather limited for the brain most probably because modulation of the Se intake only very marginally affects the brain Se status but some preliminary studies report a putative interdependency. After treating rats with a mixture of polychlorinated biphenyls, DNA damage in brain cells was increased only in rats receiving a Se-deficient diet (5 weeks, <0.05 mg/kg Se) [170]. In line with this, additional models with chemically induced brain damage showed that rather high concentrations of Se (3 weeks, 1 mg/kg scopolamine followed by 2 weeks, 1.5 mg/kg Se; 5 days, 0.625 mg/kg Se + 240 nmol/μL quinolinic acid; 7 days, 0.2 mg/kg Se followed by induction of cerebral ischemia) applied by *intraperitoneal* injections reduced the substance-induced damage to the brain [[171], [172], [173], [174]]. In an AD mouse model, animals were either fed a Se-deficient (0.1 ± 0.1 mg/kg Se), adequate (0.3 mg/kg Se) or a Se-supplemented (1.0 ± 0.1 mg/kg Se) diet for 5 months. Oxidative DNA damage was measured by the level of 8-oxodG in cortex DNA and RNA. The level of 8-oxodG was higher in brains of animals fed the Se-deficient diet compared to the normal diet. Moreover, in Se-supplemented animals significantly decreased levels of 8-oxodG as well as increased GPX activity were detected, indicating a link between the cellular redox status and DNA damage [175]. Hypothalamic selenoproteins have been recently discussed to be important mediators of energy metabolism [176]. Transgenic mice with loss of selenoprotein expression in pro-opiomelanocortin (POMC) neurons display increased oxidative stress and decreased numbers of POMC neurons in comparison to the wild type mice after being challenged with a high fat diet. Furthermore, a diminished responsiveness to leptin and insulin was observed indicating a functional decline of the respective neurons [177].

In contrast, very high Se concentrations supplied in *in vitro* studies resulted in oxidative stress as shown in human glioblastoma (10 h, 1 μM Se) [18] and glioma cells (24 h, 1–7 μM Se, effect observed ≥ 6 h, ≥ 7 μM; 10–120 min, 0–20 μM Se, effect observed ≥ 10 min, ≥ 5 μM) [178,179]. This was e.g. accompanied by an increase in PARP activity [18,180] as well as activation of p53 [180,181], and caspase-3 [180]. Whether or not these very high micromolar concentrations are achievable in the vital brain is a matter of debate. A 4 weeks lasting feeding study in mice with excessive Se concentrations (20 mg/kg Se (over 50 times the recommendation for mice)) provided as selenomethionine revealed that the Se content was further increased in all investigated tissues including the brain in comparison to the normal Se group (1 mg/kg Se (2.5 times the recommendation)). The expression of brain selenoproteins was only very moderately increased in case of GPX1/2 [164]. Ferroptotic cell death, modulated by p53 and increased concentrations of free Fe, frequently occurs in neurons which can be counteracted by GPX4 [182]. A recent study could show that a single dose of Se (1 μM) delivered into the brain indeed enhanced expression of GPX4 and additional selenoproteins and this way protected neurons from ferroptosis in a hemorrhagic stroke model [183].

In conclusion, selenoproteins are essential for proper brain function and thus brain Se levels are maintained even under Se deficient conditions. However, excessive Se concentrations, which so far have been mainly studied in cultured cells, could potentially damage neurons. Whether or not Se acts as an important modulator of genomic stability in the brain warrants further investigation.

## 7 Conclusion and outlook

TE dyshomeostasis in humans is associated with physiological, morphological, and functional changes, contributing to pathogenesis of various diseases. Epidemiological studies indicate that, dependent on the supply status, Fe, Zn, and Se deficiency occur with a moderate to high prevalence in the world population [[184], [185], [186]]. Incidence of Cu deficiency shows a lower prevalence, occurring mostly disease-induced [187], while Mn deficiency is a rare concern due to ubiquitous presence [104]. However, not only TE deficiency, but also TE overload may have deleterious consequences, especially regarding the redox-active TEs. The entire human organism is dependent on a balanced TE homeostasis, yet the brain in particular requires a well-regulated TE supply for proper functioning and protection of post-mitotic neurons. Additionally, altered maintenance of genomic stability, due to post-mitotic status of brain cells, makes the brain especially vulnerable to incur genomic instability. The current state of knowledge on the impact of these changed conditions in brain is limited, with special regard to the underlying molecular mechanisms of neuronal genomic stability. Especially the holistic view on both excessive and deficient conditions in brain is scarce. Nowadays, studies in humans, experimental animals, and cell culture examine the effect of redox-active TEs, such as Fe, Cu, and Mn, focussing on the impact of excessive conditions on oxidative stress, DDR pathways as well as DNA repair in brain, while effects of deficiency, which would be of interest for Fe, are infrequently taken into consideration (Table 2). In contrast, the impact of excessive as well as deficient conditions of Zn and Se is more comprehensively investigated. Besides effects on several antioxidant enzymes, mechanistic insights on the impact of Zn and Se on DDR as well as DNA repair of oxidative DNA damage in neurons are limited. Furthermore, more studies considering the human situation and experimental animals are required for a better understanding especially with regard to the limited transferability of experimental approaches to human physiological and pathophysiological conditions caused by TE dyshomeostasis. Besides increasing the complexity of model systems, studies should focus more on intracellular bioavailability data to strengthen their hypothesis regarding the direct influence of the TEs taken into account on enzymes, proteins, and subcellular structures. In this context, the current research area of novel methods to determine subcellular metal localisation like the nano secondary ion mass spectrometry offer a great potential [188].

**Table 2:** **Current state of literature on the impact of excessive and deficient conditions of Fe, Cu, Mn, Zn, and Se on neuronal genome stability in human, experimental animals, and*****in vitro*****studies.** Categorisation (effect: +++ strong, ++ moderate, + weak) is determined based on the currently available literature.

	Human		Experimental animals		*In vitro*	
	Deficiency	Excess	Deficiency	Excess	Deficiency	Excess
Fe		+++		+++		+++
Cu		+++		++		++
Mn		++		++		+
Zn			++		++	++
Se			++	++		++

**Fig. 3:**
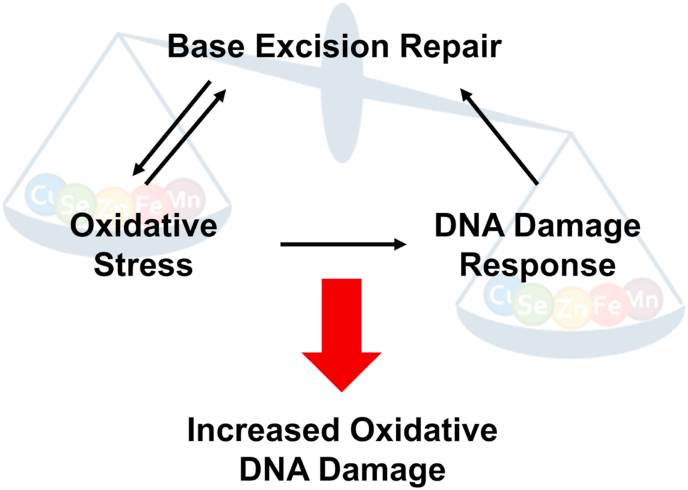
**Simplified scheme illustrating interdependence between oxidative stress, DDR, and BER in the context of TE dyshomeostasis**.

Due to increasing numbers of aged individuals, the prevention of ageing-associated neurodegenerative diseases is a highly relevant topic for today's society. In this context, the understanding of the complex influence of TE homeostasis on generation of oxidative stress, DDR pathways as well as DNA repair is of crucial interest, especially with regard to the prevention of neurodegeneration and the protection of the sensitive brain tissue from TE imbalance-induced consequences (Fig. 3).

## Funding

This research was funded by the 10.13039/501100001659German Research Foundation (DFG) Research Unit TraceAge (FOR 2558) and by the German 10.13039/501100002347Federal Ministry of Education and Research for the Competence Cluster NutriAct – Nutrition Research Berlin-Potsdam (FKZ: 01EA1806B).

## Declaration of competing interest

All authors declare that they have no conflict of interest.

## Abbreviations

8-oxodG: 8-oxo-7,8-dihydro-2′-deoxyguanosine
AD: Alzheimer's disease
AP: apurinic/apyrimidinic
APE1: apurinic/apyrimidinic endonuclease 1
APOER2: apolipoprotein E receptor 2
ATM: ataxia telangiectasia mutated serine/threonine kinase
ATP: adenosine triphosphate
BBB: blood-brain barrier
BCL2: B-cell lymphoma 2
BER: base excision repair
Ca: calcium
CAT: catalase
Cu: copper
DAergic: dopaminergic
DDR: DNA damage response
FAC: ferric ammonium citrate
Fe: iron
Fe(II): ferrous iron
Fe(III): ferric iron
FEN1: flap structure-specific endonuclease 1
GCL: glutamate cysteine ligase
GPX: glutathione peroxidase
GSH: glutathione
HF: hereditary ferritinopathy
iPSC: induced pluripotent stem cell
Mn: manganese
MUTYH: mutY homologous DNA glycosylase
NEIL: nei like DNA glycosylase
NF-κB: nuclear factor κB
NMDAR: N-methyl-d-aspartate receptor
Nrf2: nuclear factor erythroid 2-like 2 activation
OGG1: 8-oxoguanine DNA glycosylase
PARP: poly(ADP-ribose)polymerase
PARylation: poly(ADP-ribosyl)ation
PD: Parkinson's disease
PNKP: Polynucleotide Kinase 3′-Phosphatase
POLB: Polymerase beta
POMC: pro-opiomelanocortin
PSP: progressive supranuclear palsy
RONS: reactive oxygen and nitrogen species
ROS: reactive oxygen species
SAH: subarachnoid haemorrhage
Se: selenium
SELENOP: selenoprotein P
SOD: superoxide dismutase
TE: trace element
TEs: trace elements
TPEN: N,N,N′,N'-tetrakis(2-pyridylmethyl)ethylenediamine
TXNRD: thioredoxin reductase
WD: Wilson's disease
XRCC1: X-ray repair cross complementing 1
Zn: zinc
