# Supplementary material for: A matter of concern – Trace element dyshomeostasis and genomic stability in neurons
Source: Redox Biol. 2021 Jan 24;41:101877. doi: 10.1016/j.redox.2021.101877 (PMC7902532; doi:10.1016/j.redox.2021.101877)
Supplement: Multimedia component 1 [file mmc1.docx]

**A Matter of Concern – Trace Element Dyshomeostasis and Genomic Stability in Neurons**

**- Supplementary data -**

Viktoria K. Wandt^a,b#^, Nicola Winkelbeiner^a,b#^, Julia Bornhorst^b,c^, Barbara Witt^a^, Stefanie Raschke^a^, Luise Simon^a,b^, Franziska Ebert^a,b^, Anna P. Kipp^b,d¥^, Tanja Schwerdtle^a,b,e¥^*

^a^Department of Food Chemistry, Institute of Nutritional Science, University of Potsdam, Arthur-Scheunert-Allee 114-116, 14558 Nuthetal, Germany

[vwandt@uni-potsdam.de](mailto:vwandt@uni-potsdam.de), [winkelbeiner@uni-potsdam.de](mailto:winkelbeiner@uni-potsdam.de), bwitt@uni-potsdam.de, stefaras@uni-potsdam.destefaras@uni-potsdam.de, simon3@uni-potsdam.de , fraebert@uni-potsdam.de, tanja.schwerdtle@uni-potsdam.de

^b^TraceAge – DFG Research Unit on Interactions of Essential Trace Elements in Healthy and Diseased Elderly (FOR 2558), Berlin-Potsdam-Jena-Wuppertal, Germany

^c^Food Chemistry, Faculty of Mathematics and Natural Sciences, University of Wuppertal, Gaußstr. 20, 42119 Wuppertal, Germany

bornhorst@uni-wuppertal.de

^d^Department of Molecular Nutritional Physiology, Institute of Nutritional Sciences, Friedrich Schiller University Jena, Dornburger Str. 24, 07743 Jena, Germany

anna.kipp@uni-jena.de

^e^German Federal Institute for Risk Assessment (BfR), Max-Dohrn-Str. 8-10, 10589 Berlin, Germany

^#^Shared first authorship

^¥^Authors contributed equally

*Corresponding author address: Tanja Schwerdtle, Institute of Nutritional Science, University of Potsdam, Arthur-Scheunert-Allee 114-116, 14558 Nuthetal, Germany, phone: +49-33200/88-5528, fax: +49-33200/88-5573, e-mail: tanja.schwerdtle@uni-potsdam.de

**Table 1. Comprehensive list of key terms involved in literature research for this review.** Systematic literature search was conducted via PudMed as well as Web of Knowledge up to and including June 2020. The listed key terms in column 1 were combined with single or several key terms of column 2 with utilization of boolean operators, quotes and wildcards. The effects of altered TE homeostasis on neurogenesis, as well as on neural progenitor stem cells, occurring in mammalian adult brain alongside with non-self-replenishing post-mitotic neuronal cells, are not covered in this review.

| **Column 1** | **Column 2** |
| --- | --- |
| iron | oxidative stress |
| copper | reactive oxygen species |
| manganese | oxidative DNA damage |
| zinc | DNA damage response |
| selenium | p53 |
|  | DNA repair |
|  | base excision repair |
|  | PARP |
|  | genomic stability |
|  | apoptosis |
|  | neuronal death |
|  | neurodegenerative disease |
|  | neuronal cells |
|  | brain |
|  | human |
|  | *in vivo* |
|  | *in vitro* |
